# Haplotype-resolved powdery mildew resistance loci reveal the impact of heterozygous structural variation on NLR genes in *Muscadinia rotundifolia*

**DOI:** 10.1093/g3journal/jkac148

**Published:** 2022-06-13

**Authors:** Mélanie Massonnet, Amanda M Vondras, Noé Cochetel, Summaira Riaz, Dániel Pap, Andrea Minio, Rosa Figueroa-Balderas, Michael Andrew Walker, Dario Cantu

**Affiliations:** Department of Viticulture and Enology, University of California Davis, Davis, CA 95616, USA; Department of Viticulture and Enology, University of California Davis, Davis, CA 95616, USA; Department of Viticulture and Enology, University of California Davis, Davis, CA 95616, USA; Department of Viticulture and Enology, University of California Davis, Davis, CA 95616, USA; Department of Viticulture and Enology, University of California Davis, Davis, CA 95616, USA; Department of Viticulture and Enology, University of California Davis, Davis, CA 95616, USA; Department of Viticulture and Enology, University of California Davis, Davis, CA 95616, USA; Department of Viticulture and Enology, University of California Davis, Davis, CA 95616, USA; Department of Viticulture and Enology, University of California Davis, Davis, CA 95616, USA

**Keywords:** genetic resistance, haplotype phasing, nucleotide-binding leucine-rich repeat genes, genomic structural variation

## Abstract

*Muscadinia rotundifolia* cv. Trayshed is a valuable source of resistance to grape powdery mildew. It carries 2 powdery mildew resistance-associated genetic loci, *Run1.2* on chromosome 12 and *Run2.2* on chromosome 18. The purpose of this study was to identify candidate resistance genes associated with each haplotype of the 2 loci. Both haplotypes of each resistance-associated locus were identified, phased, and reconstructed. Haplotype phasing allowed the identification of several structural variation events between haplotypes of both loci. Combined with a manual refinement of the gene models, we found that the heterozygous structural variants affected the gene content, with some resulting in duplicated or hemizygous nucleotide-binding leucine-rich repeat genes. Heterozygous structural variations were also found to impact the domain composition of some nucleotide-binding leucine-rich repeat proteins. By comparing the nucleotide-binding leucine-rich repeat proteins at *Run1.2* and *Run2.2* loci, we discovered that the 2 loci include different numbers and classes of nucleotide-binding leucine-rich repeat genes. To identify powdery mildew resistance-associated genes, we performed a gene expression profiling of the nucleotide-binding leucine-rich repeat genes at *Run1.2b* and *Run2.2* loci with or without powdery mildew present. Several nucleotide-binding leucine-rich repeat genes were constitutively expressed, suggesting a role in powdery mildew resistance. These first complete, haplotype-resolved resistance-associated loci and the candidate nucleotide-binding leucine-rich repeat genes identified by this study are new resources that can aid the development of powdery mildew-resistant grape cultivars.

## Introduction

Grapevine powdery mildew (PM) is a devastating fungal disease caused by *Erysiphe necator* Schwein. (syn. *Uncinula necator*), an obligate biotrophic ascomycete that can infect all green organs of a grapevine (Gadoury *et al.* 2012). Cultivated grapevines that belong to *Vitis vinifera* (ssp. *vinifera*) are highly susceptible to PM. Fungicide sprays are applied prophylactically to control the disease but are costly (Sambucci *et al.* 2019). Natural resistance to PM exists in several wild grapes. Thirteen PM resistance-associated loci were identified in the last 2 decades (Dry *et al.* 2019; Karn *et al.* 2021). *Vitis* includes several PM-resistant species, including *Vitis* *romanetii* (Ramming *et al.* 2011; Riaz *et al.* 2011) and *Vitis* *piasezkii* (Pap *et al.* 2016), which are native to China, *V. vinifera* ssp. *sylvestris* from Central Asia (Riaz *et al.* 2020), the North American *Vitis* *cinerea* (Dalbó *et al.* 2001), and the muscadine grape, *Muscadinia rotundifolia* (Pauquet *et al.* 2001; Riaz *et al.* 2011; Feechan *et al.* 2013).

*Muscadinia rotundifolia* is closely related to *Vitis* (Small 1913). Muscadine grapes are native to the southeastern United States where they are cultivated for fruit, juice, and wine production (Olien 1990). *Muscadinia* *rotundifolia* is resistant to several diseases in addition to PM (Olmo 1971, 1986), including downy mildew, Pierce’s disease, and phylloxera. Two major genetic loci associated with PM resistance were found in *M. rotundifolia*. *Resistance to U.* *necator 1* (*Run1*), located on chromosome 12, and its alternative form, *Run1.2*, were identified in *M. rotundifolia* G52 and Trayshed, respectively (Pauquet *et al.* 2001; Riaz *et al.* 2011). Bacterial artificial chromosome sequencing of the *Run1* haplotype from *M. rotundifolia* G52 resulted in the partial reconstruction of the locus, a ∼1.2 Mb region composed of 7 TIR-NBS-LRR genes (Feechan *et al.* 2013). *Run2.1* and *Run2.2* were identified on chromosome 18 of *M. rotundifolia* Magnolia and Trayshed, respectively (Riaz *et al.* 2011). Both haplotypes of Trayshed’s *Run1.2* were associated with PM resistance and designated *Run1.2a* and *Run1.2b* (Feechan *et al.* 2015).

*Muscadinia* *rotundifolia* is an ideal partner for breeding PM-resistant grapevines that are durably resistant and require few fungicidal applications. This can be done by introgressing functionally diverse PM resistance-associated genes into *V. vinifera* (Michelmore *et al.* 2013). In wild grapes, PM resistance is associated with a programmed cell death-mediated response in infected epidermal cells. This suggests that PM resistance is based on an intracellular recognition of *E. necator*’s effectors by disease resistance (*R*) proteins that activate effector-triggered immunity (Qiu *et al.* 2015; Dry *et al.* 2019).

Most *R* genes encode nucleotide-binding leucine-rich repeat (NLR) proteins (Dubey and Singh 2018). NLRs are intracellular receptors that recognize and interact directly with pathogen-derived effectors, detect modifications in host cellular targets, or detect molecular decoys triggered by effectors (Dangl *et al.* 2013). NLR activation leads to the induction of immune responses that can restrict pathogen spread (Jones and Dangl 2006). These include calcium oscillations, a rapid burst of reactive oxygen species, extensive transcriptional reprogramming that leads to cell wall modifications, and the synthesis of pathogenesis-related proteins and antimicrobial compounds (Jones and Dangl 2006; Dangl *et al.* 2013; Kretschmer *et al.* 2019). Effector-triggered immunity is often associated with a hypersensitive response and programmed death of infected plant cells that restricts further pathogen development (Jones and Dangl 2006). NLR intracellular receptors are typically composed of 3 domains: a C-terminal leucine-rich repeat (LRR) domain, a central nucleotide-binding site domain (NBS), and a variable N-terminal domain (Meyers *et al.* 1999; McHale *et al.* 2006). The variable N-terminal domain distinguishes NLR classes. The 3 main NLR classes are the TIR-NBS-LRRs, CC-NBS-LRRs, and RPW8-NBS-LRRs; these possess N-terminal toll/interleukin-1 receptor-like (TIR), Coiled-coil (CC), and resistance to PM 8 (RPW8) domains, respectively (Meyers *et al.* 1999; Xiao *et al.* 2001; McHale *et al.* 2006; Michelmore *et al.* 2013). Only 2 TIR-NBS-LRR genes, *MrRPV1* and *MrRUN1*, have been functionally characterized in grapes (Feechan *et al.* 2013). *MrRPV1* and *MrRUN1* are at the *Run1*/*Rpv1* locus of *M. rotundifolia* G52 and confer resistance to downy mildew and PM, respectively.

The first diploid chromosome-scale genome assembly of a muscadine grape was recently published and is a valuable resource for identifying candidate PM resistance-associated NLR genes from other genetic loci in *M. rotundifolia* (Cochetel *et al.* 2021). A first analysis of the *Run1.2* locus suggested an expansion of TIR-NBS-LRR genes in *M. rotundifolia* Trayshed relative to Cabernet Sauvignon (Cochetel *et al.* 2021). Which of these TIR-NBS-LRR are involved in Trayshed’s PM resistance and to which haplotype they belong, *Run1.2a* or *Run1.2b*, is unknown. The goal of this study was to identify candidate resistance genes in each haplotype of *M. rotundifolia* Trayshed *Run1.2* and *Run2.2*. Haplotypes of Trayshed’s *R* loci were differentiated and reconstructed with deep sequencing data from 2 backcrossed *V. vinifera* lines, e6-23 (*Run1.2b*^+^) and 08391-029 (*Run2.2*^+^). Gene models in both loci were manually curated to identify the genes encoding NLRs. The 2 haplotypes of each *R* locus were compared to determine the effect of heterozygous structural variations on NLR gene content. To determine NLR genes associated with PM resistance, NLR genes’ expression in *Run1.2b* and *Run2.2* were profiled with and without PM present using RNA-sequencing (RNA-seq).

## Materials and methods

### Plant material

**Fig. 1.**
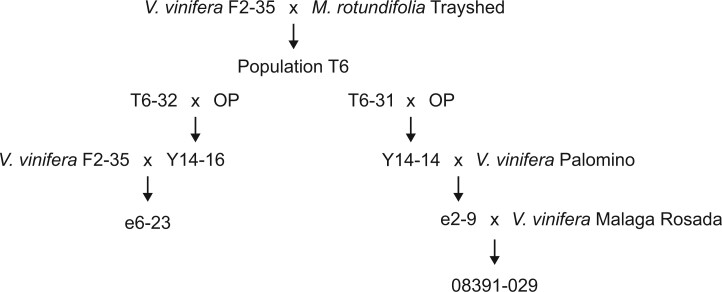
Detailed parentage of e6-23 (*Run1.2b*^+^) and 08391-029 (*Run2.2*^+^). F2-35 was produced by crossing *V. vinifera* Cabernet Sauvignon with *V. vinifera* Carignane. OP, open pollinator which is assumed *V. vinifera*.

We used 2 *V. vinifera* backcrossed lines in this study, e6-23 carrying *Run1.2b* (Feechan *et al.* 2015) and 08391-029 possessing *Run2.2* (Riaz *et al.* 2011). Both e6-23 (*Run1.2b*^+^) and 08391-029 (*Run2.2*^+^) are *V. vinifera* backcrosses derived from the T6 population series developed by Dr. Harold P. Olmo at University of California Davis (Riaz *et al.* 2011). Information about the lineage of each genotype is provided in Fig. 1. For each grape accession, 3 plants were inoculated with *E. necator* C-strain and 3 plants were mock-inoculated as described in Amrine *et al.* (2015). Two leaves from each plant were collected 1 and 5 days postinoculation (dpi) and immediately frozen in liquid nitrogen. Leaves from an individual plant were pooled together and constitute a biological replicate. Three biological replications were obtained for each treatment.

### DNA and RNA extraction, library preparation, and sequencing

Deep sequencing of e6-23 (*Run1.2b*^+^) and 08391-029 (*Run2.2*^+^) was done to distinguish the 2 haplotypes of each Trayshed’s locus. Genomic DNA was extracted from mock-inoculated leaves of e6-23 and 08391-029 and libraries were prepared as in Massonnet *et al.* (2020). Final libraries were sequenced on the Illumina HiSeqX Ten system in paired-end 150-bp reads (IDseq, Davis, CA, USA; Supplementary Table 1).

Gene expression in mock- and PM-inoculated e6-23 (*Run1.2b*^+^) and 08391-029 (*Run2.2*^+^) leaves was assessed by RNA-seq. RNA extraction and library preparation were performed as in Amrine *et al.* (2015). cDNA libraries were sequenced using an Illumina HiSeq4000 sequencer (DNA Technologies Core, University of California, Davis, CA, USA) in 50-bp single-end reads (Supplementary Table 2).

### Locus reconstruction

The *Run1.2* and *Run2.2* haplotypes were located by aligning the primers for *Run1.2*-associated markers, VMC4f3.1 and VMC8g9, and *Run2.2*-associated markers, VMC7f2 and UDV108, onto the diploid, chromosome-scale genome of *M. rotundifolia* Trayshed (Riaz *et al.* 2011; Cochetel *et al.* 2021). Whole-genome DNA sequencing reads from e6-23 (*Run1.2b*^+^) and 08391-029 (*Run2.2*^+^) were used to identify *Run1.2b* and *Run2.2* sequences. Low-quality DNA sequencing reads were removed and adapter sequences were trimmed using Trimmomatic v.0.36 (Bolger *et al.* 2014) with the following settings: LEADING:3 TRAILING:3 SLIDINGWINDOW:10:20 MINLEN:36 CROP:150. High-quality, paired-end reads were aligned onto the diploid genome of *M. rotundifolia* Trayshed (Cochetel *et al.* 2021) using BWA v.01.17 (Li and Durbin 2009) and default parameters. Reads aligning onto the reference genome with no edit distance (0 mismatch) were selected using bamtools filter v.2.5.1 (Barnett *et al.* 2011) and the tag “NM : 0.” These alignments were used as input for evaluating base coverage with genomecov (BEDTools v2.29.1; Quinlan 2014). Coverage from bases located in repetitive elements was removed using BEDTools intersect v2.29.1 (Quinlan 2014). Median coverage per 10-kb window was calculated using BEDTools map v2.29.1 (Quinlan 2014) and normalized by dividing by the sequencing coverage (Supplementary Table 1). Sequences were removed from the locus and labeled “unplaced” if DNA sequencing reads did not cover a primary contig or its alternative haplotigs. Each haplotype was fragmented into 1 kb sequences using seqkit sliding v.0.16.1 (Shen *et al.* 2016) and aligned to itself using Minimap2 v.2.12-r847-dirty (Li 2018). Sequence overlaps between contigs were removed from the locus. DNA sequencing coverage along the 4 haplotypes was manually inspected by visualizing alignments using Integrative Genomics Viewer (IGV) v.2.4.14 (Robinson *et al.* 2011). Loci were reconstructed using HaploMake.py from the HaploSync tool suite v1.0 (https://github.com/andreaminio/HaploSync; accessed: 2022 March 1).

### Haplotype sequence comparison

**Fig. 2.**
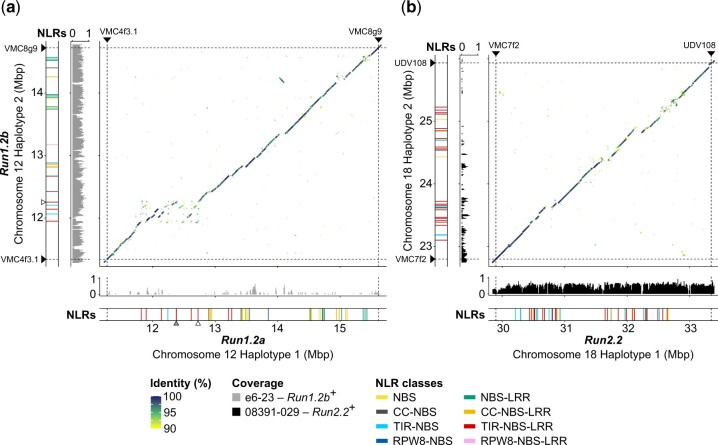
Haplotype comparison and NLR content at *Run1.2* and *Run2.2* in *M. rotundifolia* Trayshed. Whole-sequence alignments of the reconstructed haplotypes of *Run1.2* (a) and *Run2.2* (b) loci. Normalized median DNA-seq coverage per 10 kb of e6-23 (*Run1.2b*^+^) and 08391-029 (*Run2.2*^+^) on the diploid genome of *M. rotundifolia* Trayshed was used to identify *Run1.2b* and *Run2.2* on the Haplotype 2 of chromosome 12 and Haplotype 1 of chromosome 18, respectively. Only DNA-seq reads aligning perfectly on the diploid genome of *M. rotundifolia* Trayshed were used for base coverage analysis. Chromosomal position of the *Run1.2*- and *Run2.2*-associated genetic markers is indicated by black triangles and dashed lines. Chromosomal positions of the TIR-NBS-LRR genes whose predicted proteins cluster with G52’s MrRUN1 and MrRPV1 in the phylogenetic tree of Fig. 3a are indicated by gray and white triangles, respectively.

Pairwise alignments were performed using NUCmer from MUMmer v.4.0.0 (Marçais *et al.* 2018) and the --mum option. Alignments with at least 90% identity are shown in Fig. 2. Structural variants (SVs; >50 bp), SNPs, and INDELs (<50 bp) were called using show-diff and show-snps, respectively, from MUMmer v.4.0.0 (Marçais *et al.* 2018). The potential impact of SNPs on amino acid content was predicted using SnpEff v.4.3t (Cingolani *et al.* 2012).

### Annotation of NLR genes

Potential NLR genes were identified using NLR-annotator with default parameters (Steuernagel *et al.* 2020). The intron–exon structure of genes within the *R* loci was evaluated using RNA-seq alignments. RNA-seq reads from Trayshed leaves (Cochetel *et al.* 2021), e6-23 (*Run1.2b*^+^), and 08391-029 (*Run2.2*^+^) were aligned onto the diploid *M. rotundifolia* Trayshed genome using HISAT2 v.2.1.0 (Kim *et al.* 2015) and the following settings: –end-to-end –sensitive -k 50. Alignments were visualized using IGV v.2.4.14 (Robinson *et al.* 2011). Gene models were manually refined when the alignments of RNA-seq reads indicated a different intron–exon structure than the *ab initio* structural annotation.

Predicted proteins were scanned with hmmsearch from HMMER v.3.3.1 (http://hmmer.org/) and the Pfam-A Hidden Markov Models (HMM) database (El-Gebali *et al.* 2019; downloaded on 2021 January 29). Protein domains corresponding to Pfam domains including NB-ARC (PF00931.23), LRR (PF00560.34, PF07725.13, PF12799.8, PF13306.7, PF13516.7, PF13855.7), TIR (PF01582.21, PF13676.7), and RPW8 (PF05659.12), with an independent *E*-value <1.0, and an alignment covering at least 50% of the HMM were selected (Supplementary Table 3). CC domains were identified using COILS (Lupas *et al.* 1991).

### Phylogenetic analysis

Predicted NLR protein sequences from Trayshed’s *Run1.2* and *Run2.2* and G52’s *Run1/Rpv1* (Feechan *et al.* 2013) were aligned using MUSCLE (Edgar 2004) in MEGAX (Kumar *et al.* 2018). Resistance gene analogs (RGAs) from *Run1/Rpv1* (Feechan *et al.* 2013) were retrieved from GenBank using the following accession numbers: RGA1, AGC24025; RGA2, AGC24026; RGA4, AGC24027; MrRPV1 (RGA8), AGC24028; RGA9, AGC24029; MrRUN1 (RGA10), AGC24030; RGA11, and AGC24031. Phylogenetic analyses of the proteins were done with MEGAX (Kumar *et al.*, 2018) using the Neighbor-Joining method (Saitou and Nei 1987) and 1,000 bootstrap replicates.

### Gene expression analysis

Transcript abundance was evaluated with Salmon v.1.5.1 (Patro *et al.* 2017) and these parameters: --gcBias --seqBias --validateMappings. The transcriptome index file was built using a kmer size of 13, the combined transcriptomes of *M. rotundifolia* Trayshed, *V. vinifera* cv. Cabernet Sauvignon (Massonnet *et al.* 2020) and *E. necator* C-strain (Jones *et al.* 2014), and with their genomes as decoys. Quantification files were imported using an R package, tximport v.1.20.0 (Soneson *et al.* 2015). DESeq2 v.1.16.1 (Love *et al.* 2014) was used to assess differential gene expression.

## Results

### SVs between Trayshed’s *Run1.2* haplotypes affect NLR content

The boundaries of *Run1.2* were assigned by aligning the primer sequences of *Run1.2*-associated simple sequence repeats (SSR) markers on the 2 complete copies (Haplotype 1 and Haplotype 2) of chromosome 12 of *M. rotundifolia* Trayshed (Cochetel *et al.* 2021). To distinguish *Run1.2a* and *Run1.2b*, we sequenced the genome of the *V. vinifera* backcross e6-23 (*Run1.2b*^+^), into which *Run1.2b* was introgressed by crossing with *M. rotundifolia* Trayshed and backcrossing with *V. vinifera* (Fig. 1, Supplementary Tables 1 and 4). Short-sequencing reads from the *Run1.2b*^+^ accession covered and aligned perfectly (i.e. with no mismatches) to most of *Run1.2* on chromosome 12 Haplotype 2 (Supplementary Fig. 1), and coverage gaps in *Run1.2* on Haplotype 2 were complemented by coverage at *Run1.2* on Haplotype 1. This indicates that haplotype switching occurred during the assembly and phasing of Trayshed’s genome. To correct this, *Run1.2b* was reconstructed using only sequences supported with DNA sequencing reads from the *Run1.2b*^+^ accession and *Run1.2a* was reconstructed using alternative sequences (Fig. 2a). The 2 reconstructed *Run1.2* haplotypes, *Run1.2a* and *Run1.2b*, were 4.34 and 3.38 Mb long, respectively. Differences in length between the 2 haplotypes were associated with several large SVs (>50 bp). For instance, the region of *Run1.2b* from ∼12 to 12.3 Mb corresponds to a ∼800-kb region in the *Run1.2a* haplotype (Fig. 2a). In this case, their difference in length was due to several inserted sequences and duplication events in *Run1.2a*. We also found 32,704 SNPs and 7,150 INDELs between *Run1.2a* and *Run1.2b*.

To determine the effect of the heterozygous SVs and short polymorphisms on the gene content, we first refined the gene models for both *Run1.2* haplotypes. A total of 78 protein-coding genes, including 22 NLR genes, were manually annotated (Table 1). *Run1.2a* contained 253 genes and *Run1.2b* contained 189 genes, indicating that SVs affect the gene content. There were 37 and 24 NLR genes in *Run1.2a* and *Run1.2b*, respectively, with both composed primarily of CC-NBS-LRR, TIR-NBS-LRR, and NBS-LRR genes (Fig. 2a; Supplementary Table 4). SVs between haplotypes affect the protein-coding sequences of 22 NLR genes in *Run1.2a* and 9 NLR genes in *Run1.2b*. These SVs resulted in the whole duplication of 4 and 2 NLR genes in *Run1.2a* and *Run1.2b*, respectively, and the partial duplication of 3 NLR genes in *Run1.2a* (Supplementary Table 4). In addition, SVs were found to cause the loss of functionality of 4 NLR-coding genes and the hemizygosity of a CC-NBS gene in *Run1.2a* relative to *Run1.2b.* Similarly, the LRR domain of 2 NLR genes from *Run1.2b* was lost compared to *Run1.2a*. We detected 32,704 SNPs and 7,150 INDELs between the 2 *Run1.2* haplotypes (*Run1.2*a vs *Run1.2b*). Nonsynonymous SNPs were identified in 8 NLR genes in each haplotype.

**Table 1.** Sequence length, protein-coding gene content, and NLR gene content of *Run1.2* and *Run2.2* reconstructed haplotypes.

Loci	*Run1.2a*	*Run1.2b*	*Run2.2*	Chr18 Hap2
Sequence length (bp)	4,340,059	3,379,591	3,445,914	3,137,389
Protein-coding gene loci	253 (44)	189 (34)	207 (59)	179 (43)
Total NLR genes	37 (16)	24 (6)	39 (33)	29 (22)
NBS genes	3 (1)	2	6 (6)	3 (3)
CC-NBS genes	2 (1)	2	0	0
RPW8-NBS genes	2 (2)	0	0	0
TIR-NBS genes	1 (1)	2 (1)	9 (7)	3 (1)
NBS-LRR genes	8 (2)	9	4 (4)	2 (2)
CC-NBS-LRR genes	13 (6)	3 (1)	0	0
RPW8-NBS-LRR genes	0	2	0	0
TIR-NBS-LRR genes	8 (3)	4 (4)	20 (16)	21 (16)

Numbers in parentheses correspond to the genes with a structure manually refined.

### *Run2.2* is mainly composed of TIR-NBS-LRRs

A similar approach was applied to identify and reconstruct *Run2.2* in Haplotype 1 of chromosome 18 of *M. rotundifolia* Trayshed using short-sequencing reads from the genotype 08391-029 (*Run2.2*^+^; Supplementary Tables 1 and 4; Supplementary Fig. 2). The reconstructed *Run2.2* was 3.45 Mb long, slightly longer than its alternative on Haplotype 2 (3.14 Mb). We manually refined the models of 102 protein-coding genes in the 2 haplotypes, including 55 NLR genes (Table 1). More genes were annotated at *Run2.2* (207) than at its alternative (179). There were 39 NLR-coding genes at *Run2.2* and 29 NLR genes in its alternative. The 2 haplotypes were mainly composed of TIR-NBS-LRR genes, with 20 genes in *Run2.2* and 21 genes in its alternative (Supplementary Table 4). Unlike *Run1.2*, no NLR genes with a CC or RPW8 N-terminal domain were found at *Run2.2*. Interestingly, the NLR genes occurred in 2 clusters in each haplotype (Fig. 2b; Table 1).

*Run2.2* and its alternative contained 456 SVs between them, with an average length of 2.1 ± 3.6 kb. These SVs affected 21 and 16 NLR genes in *Run2.2* and its alternative, respectively. SVs were found responsible for the partial duplication of 4 and 2 NLR genes in *Run2.2* and its alternative haplotype, respectively (Supplementary Table 4). Furthermore, large deletions encompassed the complete coding sequence of 3 and 4 NLR-coding genes of *Run2.2* and its alternative haplotype, respectively. We also identified 24,128 SNPs and 5,773 INDELs between *Run2.2* and its alternative, and nonsynonymous SNPs were detected in 16 and 18 NLR genes, respectively.

### *Run1.2* and *Run2.2* loci contain distinct sets of NLRs

**Fig. 3.**
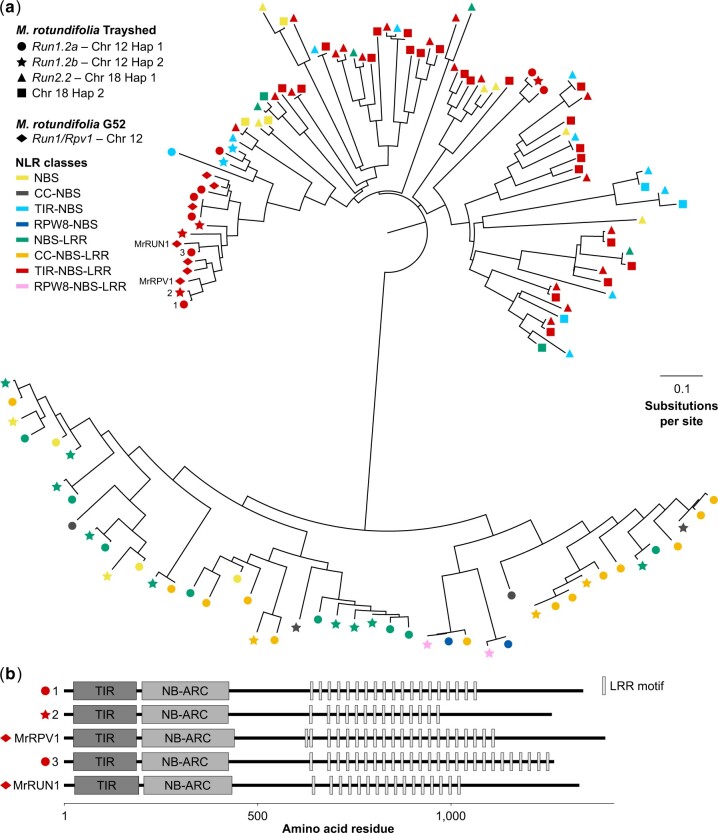
Comparison of the NLRs composing *Run1.2* and *Run2.2* in *M. rotundifolia* Trayshed. (a) Neighbor-joining clustering of the predicted protein sequences of the NLRs composing *Run1.2* and *Run2.2* haplotypes, and *Run1/Rpv1* (Feechan *et al.* 2013). (b) Domain diagram of Trayshed’s TIR-NBS-LRRs clustering with G52’s MrRUN1 and MrRPV1. Proteins are reported using same number assigned in (a**)**. LRR motifs were identified using the consensus sequence LxxLxLxx, with L indicating a leucine residue and x indicating any amino acid (Kajava and Kobe 2002).

Predicted protein sequences of the NLR genes identified in *Run1.2a*, *Run1.2b*, *Run2.2*, and *Run2.2*’s alternative on chromosome 18 Haplotype 2 were compared by constructing a phylogenic tree (Fig. 3a). In addition, we compared Trayshed’s NLRs with the TIR-NBS-LRRs at *Run1/Rpv1* in *M. rotundifolia* G52 (Barker *et al.* 2005; Feechan *et al.* 2013). *Run1/Rpv1* is the only *R* locus characterized in grapes and is an alternative version of *Run1.2*. Two distinct groups of NLRs were discovered, distinguished by the presence or absence of a TIR domain. A similar clustering pattern was observed when phylogeny was built using NBS domain sequences only (Supplementary Fig. 3), as previously observed in other plants (Seo *et al.* 2016; Prigozhin and Krasileva 2021). NLRs also tended to cluster by *R* locus, indicating an allele relationship between haplotypes for 74.4% of the NLRs (Supplementary Table 4). Regarding the TIR-containing proteins, we found the TIR-NBS-LRRs from *Run1/Rpv1* clustering with the TIR-NBS-LRR proteins from *Run1.2*. MrRPV1 from *M. rotundifolia* G52 clustered with 2 TIR-NBS-LRRs, one from each *Run1.2* haplotype, and MrRUN1 clustered with a TIR-NBS-LRR from *Run1.2a* (Fig. 3a; Supplementary Table 4). Clustering of TIR-NBS-LRRs of *Run1.2* and *Run1/Rpv1* support an allelic relationship between them. However, the number of LRR motifs in their LRR domain was different (Fig. 3b), suggesting some allelic diversity. In addition, differences in LRR domains suggest that these TIR-NBS-LRRs might be specific to different effectors and/or pathogens (McHale *et al.* 2006).

### Most of the NLR genes at *Run1.2b* and *Run2.2* are constitutively expressed

**Fig. 4.**
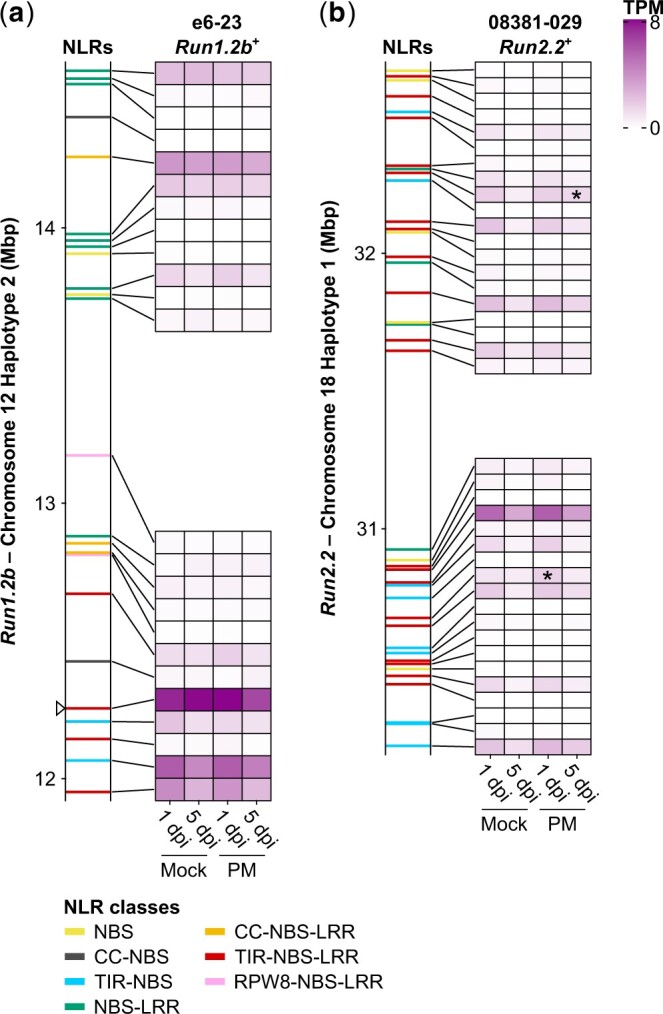
Transcript abundance of NLR genes in *Run1.2b* (a) and *Run2.2* (b). Gene expression was monitored in *Run1.2b*^+^ and *Run2.2*^+^ at 1 and 5 dpi with *E. necator* conidia (PM) or a mock solution (Mock). Transcript abundance is shown as the mean of TPM. *n* = 3. NLR genes differentially expressed in response to PM are indicated by an asterisk (*P ≤ *0.05). White triangle indicates the chromosomal position of the TIR-NBS-LRR gene whose predicted protein clusters with G52’s MrRPV1 in Fig. 3a.

Constitutive NLR gene expression is essential for disease resistance (Michelmore *et al.* 2013). To identify expressed NLR genes that are potentially responsible for PM resistance, we measured gene expression in *Run1.2b*^+^ and *Run2.2*^+^ leaves 1 and 5 dpi with either *E. necator* C-strain or a mock solution using RNA-seq (Fig. 4).

In *Run1.2b*^+^ leaves, nearly all NLR genes at *Run1.2b* (23/24) were expressed (Fig. 4a). Nine of them had an expression level higher than 1 transcript per million (TPM) in at least one condition (TPM > 1), while 15 NLR genes were expressed at a lower level (TPM ≤ 1). The most highly expressed genes in *Run1.2b* across all conditions (mean TPM > 4 TPM) included 2 TIR-NBS-LRRs and a TIR-NBS at the 5′-end of the locus. In addition, the gene with the most elevated expression was the TIR-NBS-LRR gene which predicted protein clustered with MrRPV1 in the phylogenic tree (Fig. 3; Supplementary Table 4). PM inoculation was not found to significantly impact the expression of any NLR gene composing *Run1.2b*.

In *Run2.2*^+^, we identified 11 NLR genes with a transcript abundance greater than 1 TPM, 28 lowly expressed (TPM ≤ 1), and 4 with no expression (Fig. 4b). A TIR-NBS-LRR gene at the 5′-end of *Run2.2* was the most highly expressed across conditions. Seven other TIR-NBS-LRR genes in the locus had moderate expression levels. Only 2 NLR genes at *Run2.2* were modulated in response to PM, including one at 1 dpi and another at 5 dpi.

These RNA-seq data show that most of the NLR genes composing Trayshed’s loci are constitutively expressed, although they exhibit different levels of expression in *Run1.2b*^+^ and *Run2.2*^+^ genotypes. Expressed NLR genes are candidate genes involved in PM resistance associated with *Run1.2b* and *Run2.2*. All candidate genes, their coordinates, expression, and relationship to *Run1/Rpv1*-associated NLRs are reported in Supplementary Table 4.

## Discussion

By combining a diploid assembly of Trayshed’s genome and DNA sequencing data generated from 2 backcrossed *V. vinifera* genotypes, we distinguished, phased, and reconstructed the 4 complete haplotypes of *Run1.2* and *Run2.2*. To our knowledge, this is the first report of complete, haplotype-resolved *R* loci for grapes. The *Run1*/*Rpv1* locus of *M. rotundifolia* G52 was sequenced prior, but its assembly was fragmented and haploid (Feechan *et al.* 2013). The same approach used in this study could be applied to resolve the entire *Run1/Rpv1* locus. Additional *Run1* haplotypes could be compared to better understand the evolution of the structure of the locus across muscadines and its impact on the NLR gene content.

Trayshed was previously defined as homozygous at *Run2.2* based on amplicon size (Riaz *et al.* 2011). However, *in silico* PCR of Trayshed’s genome showed 2 amplicon sizes for the UDV108 marker: 225 bp on chromosome 18 Haplotype 1 and 323 bp on chromosome 18 Haplotype 2 (Supplementary Table 4). The numerous SVs and small polymorphisms between the 2 haplotypes indicate heterozygosity between the 2 haplotypes. Although *Run2.2* locus on chromosome 18 Haplotype 1 confers resistance to PM (Riaz *et al.* 2011), evaluation of PM susceptibility and sequencing of backcrossed individuals possessing the region on chromosome 18 Haplotype 2 will be necessary to determine whether this haplotype is associated with PM resistance. Complete downy mildew resistance, *Rpv2*, was associated with the same genomic region as *Run2.2* on chromosome 18 of Trayshed (Wiedemann-Merdinoglu *et al.* 2006). If both *Run2.2* haplotypes were characterized, then resistance genes effective against *Plasmopara viticola* could be identified.

Sixty percent of the NLR genes identified among the 4 haplotypes were manually annotated (Table 1). This was made possible by correctly phasing haplotypes and highlights the necessity of meticulously dissecting complex genomic regions if candidate trait-associated genes are sought (Massonnet *et al.* 2020). SVs and short polymorphisms affecting NLR genes were discovered by comparing the haplotypes of each resistance locus. These polymorphisms could be used to design haplotype-specific markers which would accelerate the development of PM-resistant cultivars through marker-assisted selection.

The NLRs in Trayshed’s *Run1.2* and *Run2.2* loci differ. All 3 classes of NLRs, CC-NBS-LRRs, RPW8-NBS-LRRs, and TIR-NBS-LRRs were found in *Run1.2* haplotypes, but no CC or RPW8 domains were identified among NLRs at *Run2.2*. The only characterized NLR gene associated with grape PM resistance, MrRUN1, is a TIR-NBS-LRR (Feechan *et al.* 2013). Based on phylogeny, only *Run1.2a* possesses a TIR-NBS-LRR clustering with MrRUN1. This suggests that the mechanisms of PM resistance associated with Trayshed’s *Run1.2a* and the haplotype *Run1* of *M. rotundifolia* G52 might be more similar compared to *Run1.2b*. However, their number of LRR motifs differ, suggesting that they might be specific to different effectors (McHale *et al.* 2006). Comparison of the leaf transcriptome of *Run1.2a*^+^, *Run1.2b*^+^, and *Run1*^+^ accessions in response to PM would help evaluate the commonality in defense-related mechanisms between the 3 haplotypes. Furthermore, the functional characterization of the NLR genes composing Trayshed’s *R* loci would identify the NLR(s) responsible for PM resistance. This would help determine whether the NLR class is a decisive factor for grape PM resistance, and if stacking resistance genes from the 2 haplotypes of *Run1.2* enhances the level of PM resistance and/or its durability. Fine mapping and generation of sequencing data (DNA-seq and RNA-seq) from recombinants could be used to narrow down the list of candidate NLR genes to test.

Most of the NLR genes composing *Run1.2b* and *Run2.2* were expressed at a low level whether or not the pathogen was present. Constitutive low expression of NLR genes is common in plants (Michelmore *et al.* 2013; Zhang *et al.* 2020), supporting a constitutive ability to sense pathogens. In contrast, some plant NLR genes were found to be more highly expressed during pathogen infection, indicative of induction of the defense-related surveillance in response to biotic stress (Mohr *et al.* 2010; Sagi *et al.* 2017; Zhang *et al.* 2020). No NLR gene at *Run1.2b* was found differentially expressed in response to *E. necator* C-strain in the *Run1.2b*^+^ genotype, suggesting that PM resistance associated with *Run1.2b* likely relies on constitutive expression. On the other hand, 2 NLR genes were differentially expressed in response to PM in the backcrossed *V. vinifera* genotype possessing *Run2.2.* Additional expression profiling experiments could be done to determine whether the gene expression modulation of these NLR genes in response to *E. necator* plays a role in Trayshed’s PM resistance. Assessing gene expression level and transcriptional modulation of the NLR genes composing *Run1.2a* from accessions carrying only this haplotype (e.g. e1-78; Feechan *et al.* 2015) would help identify candidate NLR genes for this haplotype. In addition, it would be interesting to profile the gene expression of the candidate NLRs in different individuals carrying Trayshed’s PM-associated loci to evaluate the effect of the genetic background on NLR gene expression. Monitoring NLR gene expression in response to additional *E. necator* strains would help determine whether the NLR genes composing the 2 PM-associated loci exhibit a strain-specific gene expression and/or transcriptional modulation. Finally, the approach used in this study could be applied to other *R* loci to discover haplotype-specific markers for breeding PM-resistant cultivars.

## Data availability

Sequencing data are accessible through NCBI under the BioProject ID PRJNA780568. New genome assembly of *M. rotundifolia* Trayshed and annotation files is available at Zenodo under the DOI:10.5281/zenodo.5703495 and at www.grapegenomics.com.

Supplemental material is available at *G3* online.

## Funding

This work was partially funded by the American Vineyard Foundation grant #2017–1657, the US Department of Agriculture (USDA)-National Institute of Food and Agriculture (NIFA) Specialty Crop Research Initiative award #2017-51181-26829, the National Science Foundation (NSF) grant #1741627 and partially supported by funds to DC from the Louis P. Martini Endowment in Viticulture.

## Conflicts of interest

None declared.

## Supplementary Material

jkac148_Supplementary_Figure_1

jkac148_Supplementary_Figure_2

jkac148_Supplementary_Figure_3

jkac148_Supplementary_Table_1

jkac148_Supplementary_Table_2

jkac148_Supplementary_Table_3

jkac148_Supplementary_Table_4
