# Supplementary material for: Haplotype-resolved powdery mildew resistance loci reveal the impact of heterozygous structural variation on NLR genes in Muscadinia rotundifolia
Source: G3 (Bethesda). 2022 Jun 13;12(8):jkac148. doi: 10.1093/g3journal/jkac148 (PMC9339307; doi:10.1093/g3journal/jkac148)
Supplement: jkac148_Supplementary_Figure_1 [file jkac148_supplementary_figure_1.pdf]

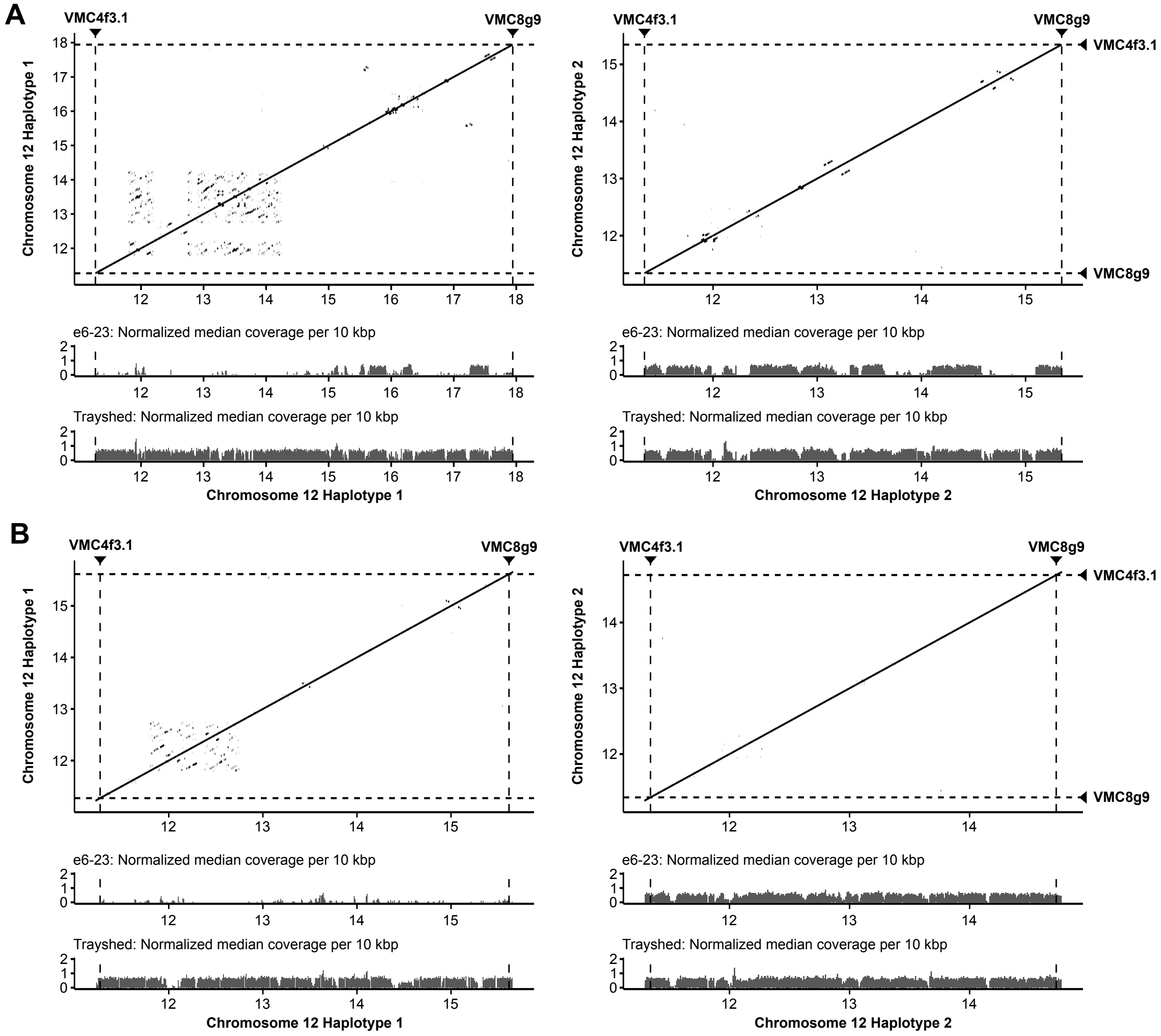

Supplementary Fig. 1: Reconstruction of *Run1.2a* and *Run1.2b* loci in *M. rotundifolia* Trayshed. Self-comparison of *Run1.2a* (chromosome 12 Haplotype 1) and *Run1.2b* (chromosome 12 Haplotype 2) loci, normalized median coverage of DNA-seq reads from e6-23 (*Run1.2b*$^{+}$) and Trayshed (*Run1.2a/b*$^{+}$) before (**A**) and after (**B**) reconstruction. Only DNA-seq reads aligning perfectly on the diploid genome of *M. rotundifolia* Trayshed were used for the base coverage analysis. Chromosomal position of the genetic markers VMC4f3.1 and WMC8g9 is indicated by black triangles and dashed lines.
