# Supplementary material for: Haplotype-resolved powdery mildew resistance loci reveal the impact of heterozygous structural variation on NLR genes in Muscadinia rotundifolia
Source: G3 (Bethesda). 2022 Jun 13;12(8):jkac148. doi: 10.1093/g3journal/jkac148 (PMC9339307; doi:10.1093/g3journal/jkac148)
Supplement: jkac148_Supplementary_Figure_2 [file jkac148_supplementary_figure_2.pdf]

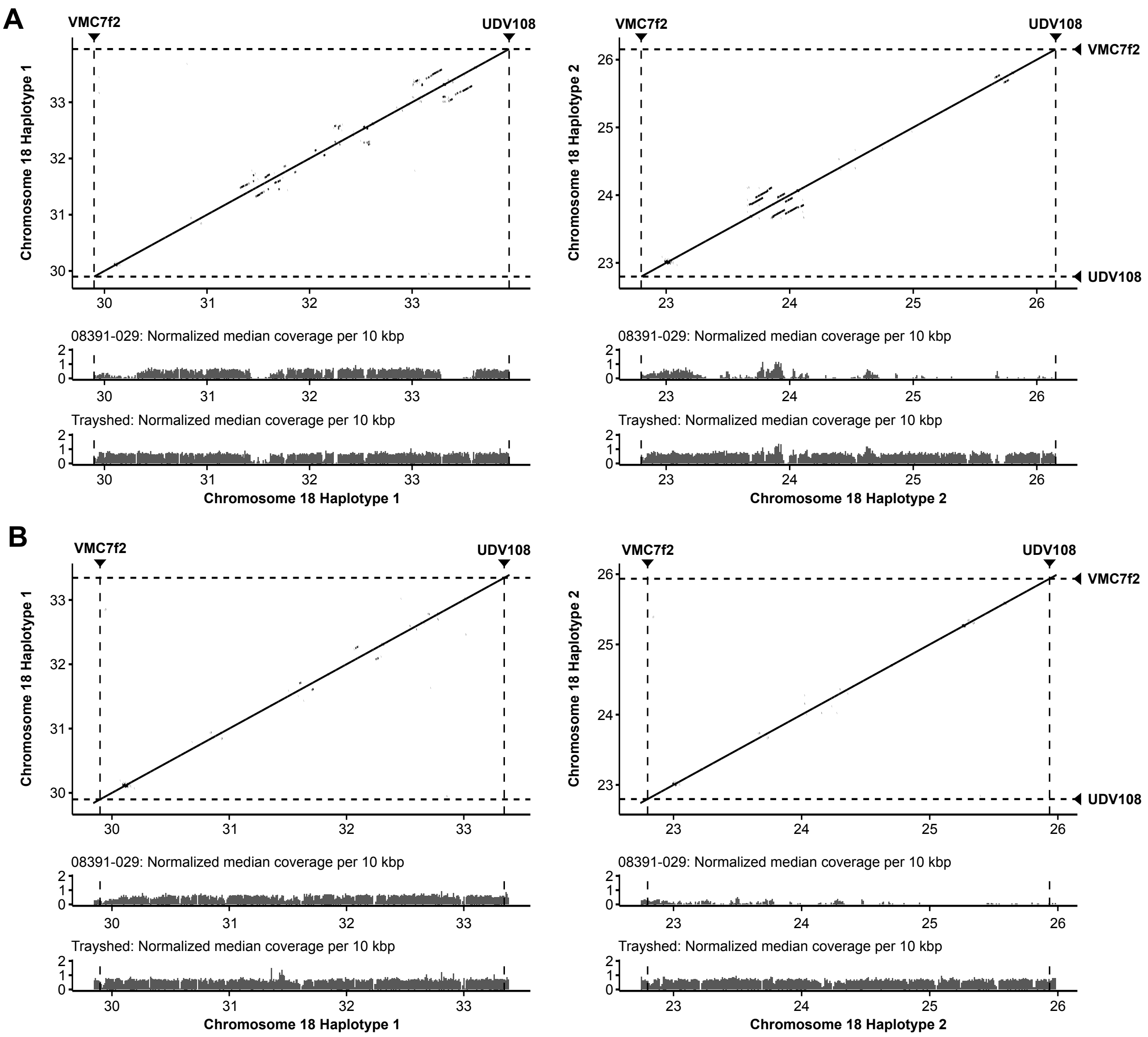

Supplementary Fig. 2: Reconstruction of *Run2.2* locus and its alternative haplotype in *M. rotundifolia* Trayshed. Self-comparison of *Run2.2* (chromosome 18 Haplotype 1) and its alternative haplotype (chromosome 18 Haplotype 2) loci, normalized median coverage of DNA-seq reads from 08391-029 (*Run2.2*$^{+}$) and Trayshed (*Run2.2*$^{+}$) before (**A**) and after (**B**) reconstruction. Only DNA-seq reads aligning perfectly on the diploid genome of *M. rotundifolia* Trayshed were used for the base coverage analysis. Chromosomal position of the genetic markers VMC7f2 and UDV108 is indicated by black triangles and dashed lines.
