# Supplementary material for: Haplotype-resolved powdery mildew resistance loci reveal the impact of heterozygous structural variation on NLR genes in Muscadinia rotundifolia
Source: G3 (Bethesda). 2022 Jun 13;12(8):jkac148. doi: 10.1093/g3journal/jkac148 (PMC9339307; doi:10.1093/g3journal/jkac148)
Supplement: jkac148_Supplementary_Figure_3 [file jkac148_supplementary_figure_3.pdf]

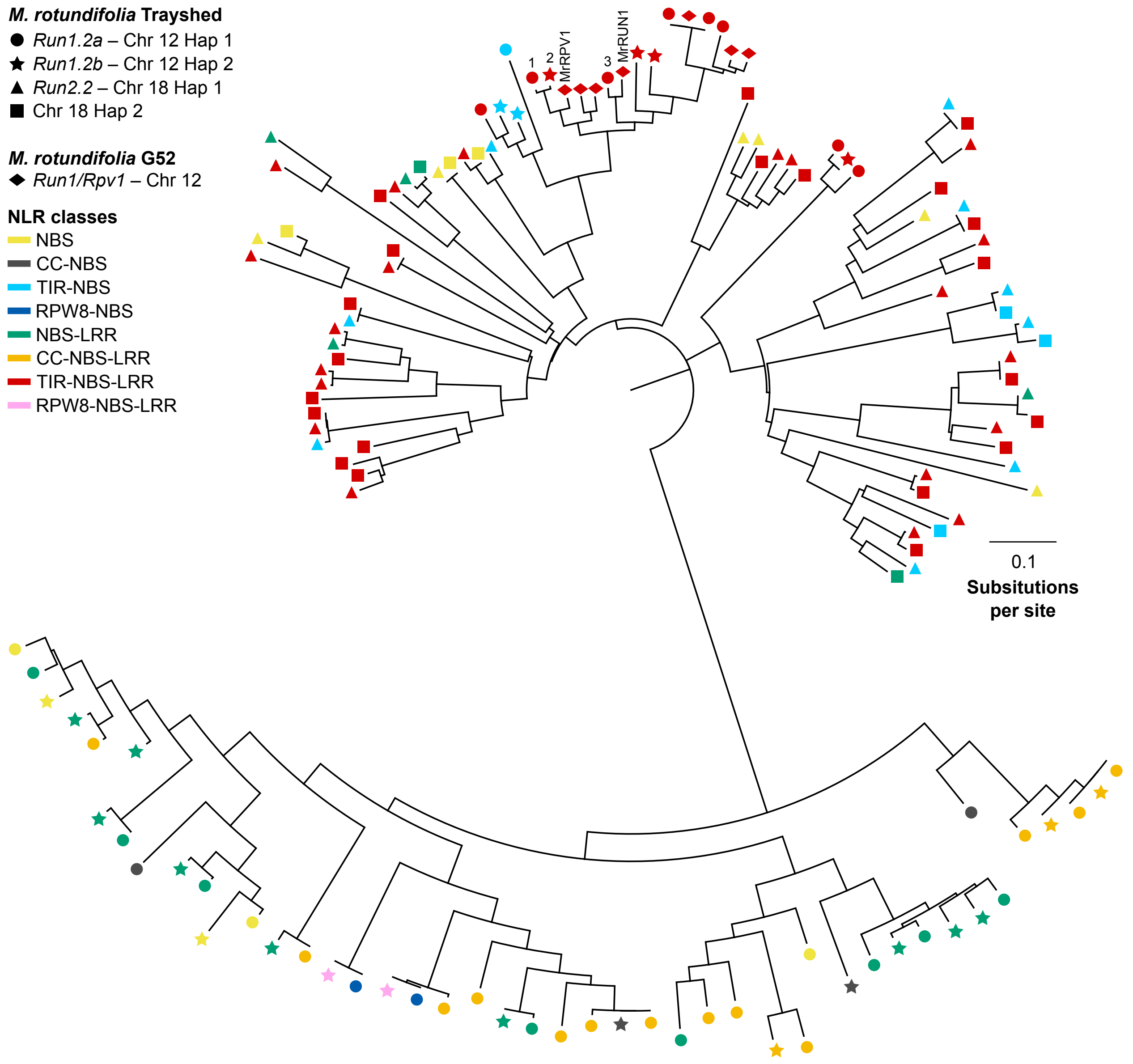

Supplementary Fig. 3: Neighbor-joining clustering of the nucleotide-binding domain of the predicted NLR protein sequences of the loci *Run1.2*, *Run2.2* and *Run1*/*Rpv1* (Feechan *et al*., 2013).
